# 1-Acetyl-6-bromo-1*H*-imidazo[4,5-*b*]pyridin-2(3*H*)-one

**DOI:** 10.1107/S1600536811017004

**Published:** 2011-05-11

**Authors:** Siham Dahmani, Youssef Kandri Rodi, Santiago V. Luis, El Mokhtar Essassi, Lahcen El Ammari

**Affiliations:** a Laboratoire de Chimie Organique Appliquée, Université Sidi Mohamed Ben Abdallah, Faculté des Sciences et Techniques, Route d’Immouzzer, BP 2202 Fès, Morocco; b Departamento de Quimica Inorganica e Organica, ESTCE, Universitat Jaume I, E-12080 Castellon, Spain; c Laboratoire de Chimie Organique Hétérocyclique URAC21, Faculté des Sciences, Université Mohammed V-Agdal, Avenue Ibn Battouta, BP 1014, Rabat, Morocco; d Laboratoire de Chimie du Solide Appliquée, Faculté des Sciences, Université Mohammed V-Agdal, Avenue Ibn Battouta, BP 1014, Rabat, Morocco

## Abstract

The two fused five- and six-membered rings in the mol­ecule of the title compound, C_8_H_6_BrN_3_O_2_, are approximately coplanar, the largest deviation from the mean plane being 0.011 (3) Å at the NH atom. The acetyl group is slightly twisted with respect to the imidazo[4,5-*b*]pyridine system, making a dihedral angle of 2.7 (2)°. In the crystal, adjacent mol­ecules are linked by inter­molecular N—H⋯N and C—H⋯O hydrogen bonds, forming infinite chains.

## Related literature

For background information on the pharmacological activities of imidazo[4,5-*b*]pyridines, see: Kale *et al.* (2009 ▶); Silverman (2004 ▶); Cristalli *et al.* (1995 ▶); Cundy *et al.* (1997 ▶); Banie *et al.* (2007 ▶); Mader (2008 ▶); Janssens *et al.* (1985 ▶); Bavetsias *et al.* (2007 ▶); Coates *et al.* (1993 ▶).
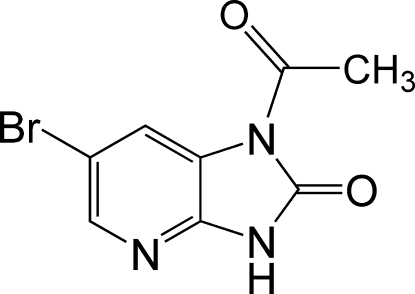


## Experimental

### 

#### Crystal data

C_8_H_6_BrN_3_O_2_
*M* *_r_* = 256.07
Triclinic, 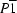

*a* = 4.8302 (15) Å
*b* = 9.645 (3) Å
*c* = 9.809 (3) Å
α = 81.542 (7)°
β = 85.735 (7)°
γ = 89.676 (8)°
*V* = 450.8 (2) Å^3^
*Z* = 2
Mo *K*α radiation
μ = 4.53 mm^−1^
*T* = 298 K
0.41 × 0.16 × 0.11 mm

#### Data collection

Bruker APEXII CCD diffractometer
Absorption correction: multi-scan (*SADABS*; Bruker, 2009 ▶) *T*_min_ = 0.423, *T*_max_ = 0.607
2559 measured reflections
1685 independent reflections
1583 reflections with *I* > 2σ(*I*)
*R*_int_ = 0.019

#### Refinement

*R*[*F* ^2^ > 2σ(*F* ^2^)] = 0.028
*wR*(*F* ^2^) = 0.074
*S* = 1.07
1685 reflections
128 parameters
H-atom parameters constrained
Δρ_max_ = 0.43 e Å^−3^
Δρ_min_ = −0.48 e Å^−3^

### 

Data collection: *APEX2* (Bruker, 2009 ▶); cell refinement: *SAINT* (Bruker, 2009 ▶); data reduction: *SAINT*; program(s) used to solve structure: *SHELXS97* (Sheldrick, 2008 ▶); program(s) used to refine structure: *SHELXL97* (Sheldrick, 2008 ▶); molecular graphics: *ORTEPIII* (Burnett & Johnson, 1996 ▶) and *ORTEP-3 for Windows* (Farrugia, 1997 ▶); software used to prepare material for publication: *SHELXL97*.

## Supplementary Material

Crystal structure: contains datablocks I, global. DOI: 10.1107/S1600536811017004/dn2682sup1.cif

Structure factors: contains datablocks I. DOI: 10.1107/S1600536811017004/dn2682Isup2.hkl

Supplementary material file. DOI: 10.1107/S1600536811017004/dn2682Isup3.cml

Additional supplementary materials:  crystallographic information; 3D view; checkCIF report

## Figures and Tables

**Table 1:** Hydrogen-bond geometry (Å, °)

*D*—H⋯*A*	*D*—H	H⋯*A*	*D*⋯*A*	*D*—H⋯*A*
N2—H2⋯N1^i^	0.86	2.02	2.877 (3)	175
C3—H3⋯O2^ii^	0.93	2.56	3.481 (3)	172

Symmetry codes: (i) 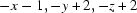; (ii) 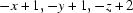.

**Fig. 1.**
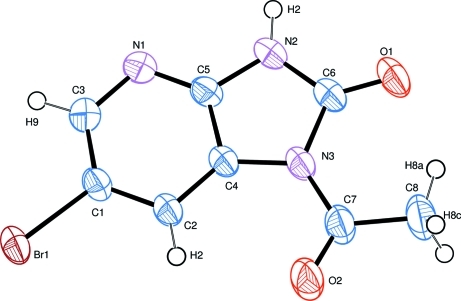
: Molecular view of the title compound with the atom-labelling scheme. Displacement ellipsoids are drawn at the 50% probability level. H atoms are represented as small circles of arbitrary radii.

**Fig. 2.**
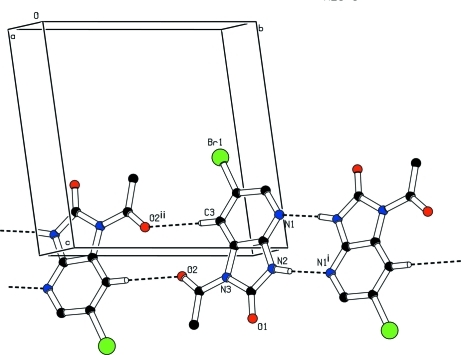
: Partial packing view showing the N-H···N and C-H···O hydrogen bonds as dashed lines. H atoms not involved in hydrogen bonding have been omitted for clarity. [Symmetry codes: (i) -x-1, -y+2, -z+2; (ii) -x+1, -y+1, -z+2].

## supplementary crystallographic information

### Comment

Imidazo[4,5-*b*]pyridines represent the major backbone of numerous medical and biochemical agents possessing different chemical and pharmacological features (Kale *et al.*, 2009; Silverman, 2004), which impart them diverse biological properties like antiviral (Cristalli *et al.*,1995; Cundy *et al.*, 1997; Banie *et al.*, 2007), and anti-inflammatory (Mader, 2008) activity. Substituted imidazo[4,5-*b*]pyridines have also been tested for their potential selective antihistamine (H1) agents (Janssens *et al.*,1985). Imidazo[4,5-*b*]pyridine derivatives were also reported as Aurora kinases (Bavetsias *et al.*, 2007) and cyclic PDE inhibitors (Coates *et al.*,1993). Importantly,imidazo[4,5-*b*]pyridine is a structural analogue of purine whose derivatives easily interact with large biomolecules such as DNA, RNA or diverse proteins *in vivo*.

The molecular plot of the crystal structure of 3-Acetyl-6-bromo-1,3-dihydro- imidazo[4,5-*b*]pyridin-2-one is shown in Fig.1. The two fused five and six-membered rings building the molecule are nearly planar with the maximum deviation from the mean plane being -0.011 (3) A ° at N2. They form a dihedral angle of 2.7 (2)° with the acetyl group. In the crystal, adjacent molecules are linked by intermolecular N—H···N and C—H···O hydrogen bonding in the way to form infinite chains as shown in Fig. 2 and Table 1.

### Experimental

To a stirred solution of 6-bromo-1,3-dihydro-imidazo[4,5 - b-]pyridin-2-one (0.2 g; 93.4 mmol), K_2_CO_3_ (0.38 g; 2.8 mmol), and tetra *n*-butyl ammonium bromide (0.03 g; 9.34 10–5 mol) in DMF, acetyl chloride (0.08 ml; 1.12 mmol) was added dropwise. The mixture was heated under reflux for 24 h. After completion of reaction (monitored by TLC), the salt was filtered and the solvent was removed under reduced pressure. The resulting residue was purified by column chromatography on silica gel using (ethylacetate/hexane) (1/1) as eluent. Crystals were isolated after the solvent was allowed to evaporate.

### Refinement

H atoms were located in a difference map and treated as riding with C—H = 0.93 Å for all aromatic H atoms and 0.96 Å for the methyl with *U*_iso_(H) = 1.2 *U*_eq_ and *U*_iso_(H) = 1.5 *U*_eq_ for the aromatic and methyl respectively.

### Crystal data

**Table:**

C_8_H_6_BrN_3_O_2_	*Z* = 2
*M**_r_* = 256.07	*F*(000) = 252
Triclinic, *P*1	*D*_x_ = 1.887 Mg m^−^^3^
Hall symbol: -P 1	Melting point: 507 K
*a* = 4.8302 (15) Å	Mo *K*α radiation, λ = 0.71073 Å
*b* = 9.645 (3) Å	Cell parameters from 1685 reflections
*c* = 9.809 (3) Å	θ = 2.1–26.0°
α = 81.542 (7)°	µ = 4.53 mm^−^^1^
β = 85.735 (7)°	*T* = 298 K
γ = 89.676 (8)°	Fiber, colourless
*V* = 450.8 (2) Å^3^	0.41 × 0.16 × 0.11 mm

### Data collection

**Table:**

Bruker APEXII CCD diffractometer	1685 independent reflections
Radiation source: fine-focus sealed tube	1583 reflections with *I* > 2σ(*I*)
graphite	*R*_int_ = 0.019
φ and ω scans	θ_max_ = 26.0°, θ_min_ = 2.1°
Absorption correction: multi-scan (*SADABS*; Bruker, 2009)	*h* = −5→5
*T*_min_ = 0.423, *T*_max_ = 0.607	*k* = −11→11
2559 measured reflections	*l* = 0→12

### Refinement

**Table:**

Refinement on *F*^2^	Primary atom site location: structure-invariant direct methods
Least-squares matrix: full	Secondary atom site location: difference Fourier map
*R*[*F*^2^ > 2σ(*F*^2^)] = 0.028	Hydrogen site location: inferred from neighbouring sites
*wR*(*F*^2^) = 0.074	H-atom parameters constrained
*S* = 1.07	*w* = 1/[σ^2^(*F*_o_^2^) + (0.0374*P*)^2^ + 0.2048*P*] where *P* = (*F*_o_^2^ + 2*F*_c_^2^)/3
1685 reflections	(Δ/σ)_max_ = 0.001
128 parameters	Δρ_max_ = 0.43 e Å^−^^3^
0 restraints	Δρ_min_ = −0.48 e Å^−^^3^

### Special details

**Table:**

Geometry. All esds (except the esd in the dihedral angle between two l.s. planes) are estimated using the full covariance matrix. The cell esds are taken into account individually in the estimation of esds in distances, angles and torsion angles; correlations between esds in cell parameters are only used when they are defined by crystal symmetry. An approximate (isotropic) treatment of cell esds is used for estimating esds involving l.s. planes.
Refinement. Refinement of *F*^2^ against ALL reflections. The weighted *R*-factor *wR* and goodness of fit *S* are based on *F*^2^, conventional *R*-factors *R* are based on *F*, with *F* set to zero for negative *F*^2^. The threshold expression of *F*^2^ > 2σ(*F*^2^) is used only for calculating *R*-factors(gt) *etc*. and is not relevant to the choice of reflections for refinement. *R*-factors based on *F*^2^ are statistically about twice as large as those based on *F*, and *R*- factors based on ALL data will be even larger.

### Fractional atomic coordinates and isotropic or equivalent isotropic displacement parameters (Å^2^)

**Table:**

	*x*	*y*	*z*	*U*_iso_*/*U*_eq_	
C1	−0.0666 (6)	0.9228 (3)	0.7492 (3)	0.0412 (6)	
H1	−0.0912	0.9789	0.6654	0.049*	
C2	0.1373 (5)	0.8216 (3)	0.7536 (2)	0.0371 (5)	
C3	0.1881 (5)	0.7340 (3)	0.8759 (2)	0.0377 (5)	
H3	0.3253	0.6658	0.8796	0.045*	
C4	0.0199 (5)	0.7562 (3)	0.9904 (2)	0.0355 (5)	
C5	−0.1838 (5)	0.8602 (3)	0.9766 (3)	0.0372 (5)	
C6	−0.2202 (6)	0.7636 (3)	1.2009 (3)	0.0435 (6)	
C7	0.1715 (7)	0.5870 (3)	1.1871 (3)	0.0466 (6)	
C8	0.1168 (8)	0.5301 (3)	1.3357 (3)	0.0588 (8)	
H8A	0.2348	0.4510	1.3590	0.088*	
H8B	−0.0740	0.5012	1.3534	0.088*	
H8C	0.1541	0.6013	1.3909	0.088*	
N1	−0.2322 (5)	0.9436 (2)	0.8618 (2)	0.0426 (5)	
N2	−0.3244 (5)	0.8613 (3)	1.1034 (2)	0.0435 (5)	
H2	−0.4601	0.9164	1.1191	0.052*	
N3	−0.0003 (5)	0.6957 (2)	1.1302 (2)	0.0395 (5)	
O1	−0.2966 (5)	0.7398 (2)	1.3223 (2)	0.0595 (6)	
O2	0.3528 (6)	0.5462 (3)	1.1138 (2)	0.0757 (8)	
Br1	0.35286 (6)	0.80290 (3)	0.58804 (2)	0.04632 (13)	

### Atomic displacement parameters (Å^2^)

**Table:**

	*U*^11^	*U*^22^	*U*^33^	*U*^12^	*U*^13^	*U*^23^
C1	0.0412 (14)	0.0477 (14)	0.0338 (13)	0.0047 (11)	−0.0012 (10)	−0.0033 (10)
C2	0.0392 (14)	0.0439 (13)	0.0276 (11)	−0.0007 (10)	0.0057 (10)	−0.0072 (9)
C3	0.0387 (14)	0.0428 (12)	0.0310 (12)	0.0059 (10)	0.0035 (10)	−0.0064 (10)
C4	0.0369 (13)	0.0405 (12)	0.0283 (11)	0.0025 (10)	0.0032 (9)	−0.0050 (9)
C5	0.0330 (13)	0.0449 (13)	0.0341 (12)	0.0037 (10)	0.0031 (10)	−0.0100 (10)
C6	0.0405 (15)	0.0523 (15)	0.0366 (14)	0.0057 (11)	0.0085 (11)	−0.0088 (11)
C7	0.0598 (18)	0.0451 (14)	0.0325 (13)	0.0098 (12)	0.0069 (12)	−0.0034 (10)
C8	0.078 (2)	0.0572 (17)	0.0349 (15)	0.0131 (15)	0.0123 (14)	0.0039 (12)
N1	0.0397 (13)	0.0504 (12)	0.0371 (12)	0.0090 (10)	−0.0002 (9)	−0.0059 (9)
N2	0.0382 (12)	0.0550 (13)	0.0359 (12)	0.0105 (10)	0.0076 (9)	−0.0074 (9)
N3	0.0417 (12)	0.0461 (11)	0.0287 (10)	0.0075 (9)	0.0083 (8)	−0.0045 (8)
O1	0.0640 (14)	0.0745 (14)	0.0354 (11)	0.0152 (11)	0.0188 (9)	−0.0049 (9)
O2	0.0976 (19)	0.0822 (16)	0.0386 (11)	0.0532 (15)	0.0185 (11)	0.0060 (10)
Br1	0.0522 (2)	0.0565 (2)	0.02805 (16)	0.00477 (12)	0.00836 (11)	−0.00472 (11)

### Geometric parameters (Å, °)

**Table:**

C1—N1	1.354 (3)	C6—O1	1.209 (3)
C1—C2	1.381 (4)	C6—N2	1.363 (4)
C1—H1	0.9300	C6—N3	1.433 (3)
C2—C3	1.398 (4)	C7—O2	1.193 (4)
C2—Br1	1.894 (2)	C7—N3	1.409 (4)
C3—C4	1.379 (3)	C7—C8	1.486 (4)
C3—H3	0.9300	C8—H8A	0.9600
C4—C5	1.401 (4)	C8—H8B	0.9600
C4—N3	1.406 (3)	C8—H8C	0.9600
C5—N1	1.319 (3)	N2—H2	0.8600
C5—N2	1.374 (3)		
			
N1—C1—C2	122.6 (2)	N2—C6—N3	105.9 (2)
N1—C1—H1	118.7	O2—C7—N3	118.5 (2)
C2—C1—H1	118.7	O2—C7—C8	123.7 (3)
C1—C2—C3	122.0 (2)	N3—C7—C8	117.8 (2)
C1—C2—Br1	118.30 (19)	C7—C8—H8A	109.5
C3—C2—Br1	119.66 (19)	C7—C8—H8B	109.5
C4—C3—C2	115.1 (2)	H8A—C8—H8B	109.5
C4—C3—H3	122.5	C7—C8—H8C	109.5
C2—C3—H3	122.5	H8A—C8—H8C	109.5
C3—C4—C5	119.2 (2)	H8B—C8—H8C	109.5
C3—C4—N3	134.3 (2)	C5—N1—C1	115.0 (2)
C5—C4—N3	106.5 (2)	C6—N2—C5	110.9 (2)
N1—C5—N2	125.6 (2)	C6—N2—H2	124.6
N1—C5—C4	126.0 (2)	C5—N2—H2	124.6
N2—C5—C4	108.4 (2)	C4—N3—C7	124.2 (2)
O1—C6—N2	127.0 (3)	C4—N3—C6	108.4 (2)
O1—C6—N3	127.1 (3)	C7—N3—C6	127.4 (2)
			
N1—C1—C2—C3	−0.5 (5)	N1—C5—N2—C6	−178.9 (3)
N1—C1—C2—Br1	179.6 (2)	C4—C5—N2—C6	0.7 (3)
C1—C2—C3—C4	0.1 (4)	C3—C4—N3—C7	0.3 (5)
Br1—C2—C3—C4	−179.98 (19)	C5—C4—N3—C7	−179.4 (3)
C2—C3—C4—C5	0.5 (4)	C3—C4—N3—C6	−179.8 (3)
C2—C3—C4—N3	−179.2 (3)	C5—C4—N3—C6	0.5 (3)
C3—C4—C5—N1	−0.8 (4)	O2—C7—N3—C4	2.8 (5)
N3—C4—C5—N1	178.9 (3)	C8—C7—N3—C4	−177.5 (3)
C3—C4—C5—N2	179.5 (2)	O2—C7—N3—C6	−177.1 (3)
N3—C4—C5—N2	−0.7 (3)	C8—C7—N3—C6	2.6 (5)
N2—C5—N1—C1	−179.9 (3)	O1—C6—N3—C4	−179.6 (3)
C4—C5—N1—C1	0.5 (4)	N2—C6—N3—C4	0.0 (3)
C2—C1—N1—C5	0.2 (4)	O1—C6—N3—C7	0.3 (5)
O1—C6—N2—C5	179.1 (3)	N2—C6—N3—C7	179.8 (3)
N3—C6—N2—C5	−0.4 (3)		

### Hydrogen-bond geometry (Å, °)

**Table:**

*D*—H···*A*	*D*—H	H···*A*	*D*···*A*	*D*—H···*A*
N2—H2···N1^i^	0.86	2.02	2.877 (3)	175.
C3—H3···O2^ii^	0.93	2.56	3.481 (3)	172.

Symmetry codes: (i) −*x*−1, −*y*+2, −*z*+2; (ii) −*x*+1, −*y*+1, −*z*+2.
